# Supplementary material for: Mechanisms governing the pioneering and redistribution capabilities of the non-classical pioneer PU.1
Source: Nat Commun. 2020 Jan 21;11:402. doi: 10.1038/s41467-019-13960-2 (PMC6972792; doi:10.1038/s41467-019-13960-2)
Supplement: Supplementary file 7 — Source data [file 41467_2019_13960_MOESM7_ESM.zip › Source_Data/FigureS2/FigureS2B_MotifScanOutput/KmeansCluster4/homerResults/motif17.similar.html]

motif17

## Information for motif17

C
G
T
A
G
A
T
C
G
T
C
A
A
G
T
C
G
A
C
T
A
C
G
T
A
G
C
T
G
C
A
T
T
C
G
A
G
T
C
A
C
G
T
A
A
G
T
C
G
T
A
C
C
T
G
A
  
Reverse Opposite:  

A
G
C
T
A
C
T
G
T
A
C
G
G
C
A
T
A
C
G
T
A
C
G
T
C
G
T
A
C
T
G
A
G
T
C
A
C
T
G
A
C
T
A
G
A
C
G
T
C
T
A
G
A
C
G
T
  

|  |  |
| --- | --- |
| p-value: | 1e-55 |
| log p-value: | -1.273e+02 |
| Information Content per bp: | 1.791 |
| Number of Target Sequences with motif | 95.0 |
| Percentage of Target Sequences with motif | 2.10% |
| Number of Background Sequences with motif | 60.1 |
| Percentage of Background Sequences with motif | 0.14% |
| Average Position of motif in Targets | 97.9 +/- 27.9bp |
| Average Position of motif in Background | 104.1 +/- 73.8bp |
| Strand Bias (log2 ratio + to - strand density) | 0.0 |
| Multiplicity (# of sites on avg that occur together) | 1.00 |
| Motif File: | file (matrix) reverse opposite |

### Similar de novo motifs found

|  |  |  |  |  |  |  |  |
| --- | --- | --- | --- | --- | --- | --- | --- |
| Rank | Match Score | Redundant Motif | P-value | log P-value | % of Targets | % of Background | Motif file |
| 1 | 0.877 | A G T C C G T A A G T C C T G A A G T C A G C T A C G T A C G T G C A T C T G A C G T A C G T A G T A C | 1e-49 | -114.145568 | 1.73% | 0.09% | motif file (matrix) |
| 2 | 0.914 | G T C A A G T C T G C A A G T C G A C T A C G T A G C T G C A T C T G A C G T A C G T A A G T C | 1e-47 | -109.256116 | 1.84% | 0.13% | motif file (matrix) |
| 3 | 0.794 | A G T C C T G A A G T C C T G A A G T C A C G T A C G T A G C T G C A T C G T A C G T A | 1e-38 | -88.355073 | 1.79% | 0.19% | motif file (matrix) |
| 4 | 0.829 | C G T A A G T C C T G A A G T C A C G T A C G T A C G T G C A T C G T A C G T A | 1e-28 | -66.538690 | 1.84% | 0.32% | motif file (matrix) |
| 5 | 0.759 | A C T G A C G T A C G T A C G T C G T A C G T A C G T A C G T A A C T G | 1e-17 | -40.056327 | 1.48% | 0.36% | motif file (matrix) |
